# A Novel Long Noncoding RNA, Lnc-OAD, Is Required for Bone Morphogenetic Protein 2- (BMP-2-) Induced Osteoblast Differentiation

**DOI:** 10.1155/2021/6697749

**Published:** 2021-03-16

**Authors:** Zonggui Wang, Yanfang Zhou, Zhong Dai, Xiuju Chen, Chuyu Li, Zhanying Lin, Huixing Wu, Siqi Li, Changqing Zuo

**Affiliations:** ^1^Department of Biochemistry and Molecular Biology, Guangdong Provincial Key Laboratory of Medical Molecular Diagnostics, Guangdong Medical University, Dongguan, China; ^2^Department of Pathophysiology, Guangdong Medical University, Dongguan, China; ^3^School of Pharmacy, Guangdong Medical University, Dongguan, China; ^4^Guangdong Key Laboratory for Research and Development of Natural Drugs, Zhanjiang, China; ^5^Key Laboratory of Traditional Chinese Medicine and New Pharmacy Development, Guangdong Medical University, Dongguan, China

## Abstract

Long noncoding RNAs (lncRNAs) play very important roles in cell differentiation. Our recent study has demonstrated that a novel lncRNA named lnc-OAD modulated 3T3-L1 adipocyte differentiation. In the present study, we examined the roles of lnc-OAD in bone morphogenetic protein 2- (BMP-2-) induced osteoblast differentiation. Lnc-OAD expression was increased during BMP-2-induced osteoblast differentiation in C3H10T1/2 mesenchymal stem cells and MC3T3-E1 preosteoblast cells. Knockdown of lnc-OAD expression by specific siRNA remarkably decreased early osteoblast differentiation. In addition, stable knockdown of lnc-OAD by lentivirus vector also significantly inhibited late osteoblast differentiation and matrix mineralization in vitro. Conversely, stably overexpressed lnc-OAD with lentiviral vector accelerated osteoblast differentiation. Mechanistically, knockdown of lnc-OAD reduced significantly the phosphorylation of AKT and the expression of Osterix induced by BMP-2, while overexpression of lnc-OAD enhanced the phosphorylation of AKT and the expression of Osterix. Taken together, our study suggests that lnc-OAD plays an important role in modulating BMP-2-induced osteoblast differentiation via, at least in part, regulating the AKT-Osterix signaling axis.

## 1. Introduction

Osteoblast commitment and differentiation from mesenchymal stem cells (MSCs) is a complex multistep process controlled by intricate signaling cascades and cellular molecules [1, 2]. In the osteoblast commitment and differentiation process, bone morphogenetic protein (BMP) superfamily functions as a powerful and effective signal for osteoblast inducing [3]. Various BMPs, including BMP-2, BMP-4, BMP-7, and BMP-9, have been investigated extensively and proved to induce osteoblast differentiation [4, 5]. In the past decades, a number of signaling pathways such as PI3K-AKT, MAPK, BMP-Smad, and WNT; some key transcription factors; and noncoding RNAs have been identified to be involved in BMP-2-induced osteoblast differentiation [6–9]. However, the molecular switches that modulate osteoblast differentiation, especially BMP-induced osteoblast differentiation, have not been fully elucidated. Identification of additional novel factors may provide better insights into the precise regulation of osteoblast differentiation.

Long noncoding RNAs (lncRNAs), a novel class of RNA with no significant protein-coding potential, have been recently identified as key mediators of many biological processes though diverse mechanisms such as transcription regulation and epigenetic chromatin modification [10]. In recent years, more and more studies have shown that lncRNAs were involved in osteoblast differentiation [11]. For example, Lnc-AK045490 inhibits osteoblast differentiation of mesenchymal stem cells (BMSCs) though suppression *β*-catenin/TCF1/Runx2 signaling [12]. LncRNA ODIR1 regulates the osteoblast differentiation of hUC-MSCs through the FBXO25/H2BK120ub/H3K4me3/OSX axis [13]. HOTAIR inhibits, whereas KCNQ1OT1 promotes osteogenic differentiation via Wnt/*β*-catenin signaling pathway [14, 15]. LncRNA UCA1 affects osteoblast differentiation by regulating BMP-2 expression [16]. Although the functions of above lncRNAs have been identified in osteoblast differentiation in vitro or in vivo, the potential functions of novel lncRNAs modulating osteoblast differentiation are still waiting for elucidating. In particular, whether the long noncoding RNAs regulated by BMPs affect osteoblast differentiation needs to be further clarified.

Recently, we have demonstrated that the lnc-OAD, one newly identified lncRNA transcribed from mouse 1700018A04Rik gene, modulated adipogenesis [17]. Additionally, our previous studies on microarray analysis also showed that BMP-2 upregulated the expression of lnc-OAD in mouse C3H10T1/2 mesenchymal stem cells [18]. Here, we confirmed that lnc-OAD regulated BMP-2-mediated osteoblast differentiation. Mechanistically, we revealed that AKT-Osterix signaling axis, at least in part, was regulated by lnc-OAD.

## 2. Materials and Methods

### 2.1. Materials and Reagents

Mouse C3H10T1/2 mesenchymal stem cells and mouse MC3T3-E1 preosteoblast cells were obtained from Chinese Academy of Science Cell Bank (Shanghai, China). Recombinant human BMP-2 (rhBMP-2, Humankine®, HZ-1128) was provided by Proteintech Group, Inc. (Wuhan, China). TRIzol reagent and Lipofectamine RNAiMAX were obtained from Thermo Fisher Scientific (Waltham, MA, USA). p-nitrophenyl phosphate (p-NPP), ascorbic acid, and *β*-glycerophosphate were purchased from Sigma-Aldrich (St. Louis, MO, USA). Antibody sources were as follows: p-ERK1/2 (Thr202/Tyr204, #4377), ERK1/2 (#4695), p38 (#9212), p-p38 (Thr180/Tyr182, #9215), and Runx2 (#12556) were obtained from Cell Signaling Technology (Danvers, MA USA); AKT (ab32505), p-AKT (Ser473, ab81283), and ALPL (ab108337) were purchased from Abcam (Cambridge, MA, USA); Osteocalcin (sc-390877), and Osterix (sc-393325) were provided by Santa Cruz Biotechnologies (Santa Cruz, CA, USA); *β*-actin (AF5001), HRP-labeled goat anti-mouse (A0216), and anti-rabbit (A0208) IgG were obtained from Beyotime Biotechnology (Shanghai, China).

### 2.2. Cell Culture and Osteoblast Differentiation

C3H10T1/2 cells were cultured in Dulbecco's modified Eagle's medium (DMEM) containing 10% fetal bovine serum (FBS, Cellmax, Beijing, China). MC3T3-E1 cells were cultured in a-MEM medium without ascorbic acid (A1049001, Thermo Fisher Scientific, Waltham, MA, USA) containing 10% FBS. Both cells were maintained at 37°C in a humidified atmosphere of 5% CO2 in air. To induce early osteoblast differentiation, cells grown to 90% confluence were replaced with differentiation medium containing DMEM (for C3H10T1/2) or a-MEM (for MC3T3-E1), 2.5% FBS, and 100 or 200 ng/mL rhBMP-2, and the differentiation medium was changed every 2-3 days. To induce terminal osteoblast differentiation and mineralization, cells were cultured in osteoblast mineralization medium containing 2.5% FBS, 100 ng/mL rhBMP-2, 50 *μ*g/mL ascorbic acid, and 10 mM *β*-glycerophosphate.

### 2.3. Alkaline Phosphatase (ALP) Activity Assay and ALP Staining

Levels of early osteoblast differentiation were determined by alkaline phosphatase (ALP) activity assay and ALP staining analysis. For ALP activity assay, cells were cultured in 24-well plates and induced for early osteoblast differentiation. Cell extracts were prepared with CytoBuster™ protein extraction reagent and centrifuged at 15,000g for 5 minutes. Then, 10 *μ*L cell extracts were added and incubated with 100 *μ*L of p-nitrophenyl phosphate (p-NPP) for 30 min and stopped by adding 100 *μ*L of 1 N NaOH, and then, absorbance was read at 405 nm. The protein content of cell extracts was measured by the BCA method. The OD405 values was standardized by protein content, and then, the relative ALP activity was presented as fold change to the control group. For ALP staining, cells were washed with PBS twice, fixed with 70% ethanol for 20 min. After washing three times with PBS, cells were incubated with a mixture of 0.1 mg/mL napthol AS-MX phosphate and 0.6 mg/mL Fast Blue BB salt for 30 min at room temperature.

### 2.4. Alizarin Red-S Staining and Quantification

MC3T3-E1 cells were seeded into 24-well plates, and when they reached about 90% confluence, the osteoblast mineralization medium was added to induce mineralization. On day 10, cells were fixed with 70% ethanol for 20 min and stained with 0.5% Alizarin red S solution (AR-S, pH 4.2) for 10 min at room temperature. For quantification of staining, the AR-S stain was extracted using 10% cetylpyridinium chloride for 15 min and quantified by measuring its absorbance at 540 nm. Relative AR-S stain was calculated as fold change of the control group.

### 2.5. RNA Interference

Small interfering RNAs (siRNAs) specific for mouse lnc-OAD and negative control siRNAs (si-NC) were synthesized by Shanghai GenePharma Co. Ltd. (Shanghai, China). In order to prolong the time of siRNA stabilization, siRNAs were chemically modified with 2′-O-methylation (2′-OMe). The nucleotide sequences for mouse lnc-OAD siRNA were si-lnc-OAD #1: sense, 5′-CCAGGUGUGUCCUGUGAUUTT-3′; antisense, 5′-AAUCACAGGACACACCUGGTT-3′; si-lnc-OAD #2: sense, 5′-CCUACUGAAGCAUGGCUAUTT-3′; antisense, 5′-AUAGCCAUGCUUCAGUAGGTT-3′; si-NC: sense, 5′-UUCUCCGAACGUGUCACGUTT-3′, antisense, 5′-ACGUGACACGUUCGGAGAATT-3′. After cells were grown to 40–60% confluence, siRNA duplexes with either lnc-OAD siRNA or si-NC were transfected into the cells using Lipofectamine RNAiMAX according to the manufacturer's protocol.

### 2.6. Lentiviral Infection and Generating Stable Cell Lines

Lentivirus particles for shRNA-mediated knockdown of lnc-OAD (sh-Lnc-OAD) and negative control sh-NC were provided by Shanghai Genechem Co. Ltd. (Shanghai, China). The shRNA sequences were designed, and stable lnc-OAD knockdown cells were generated as our previously described [17]. For generating cells with stable overexpression of lnc-OAD (LV-lnc-OAD) or control GFP (LV-Con), lentiviral vector carrying lnc-OAD gene (pLV[ncRNA]-EGFP:T2A:Puro-EF1A> {m1700018A04Rik[NR_029439.1]}) or control vector (pLV[Exp]-EGFP:T2A:Puro-Null) was constructed and packaged by Cyagen Biosciences Inc (Guangzhou, China). Then, MC3T3-E1 and C3H10T1/2 cells were infected with lentivirus particles and selected using the puromycin (8 *μ*g/mL for MC3T3-E1 and 3 *μ*g/mL for C3H10T1/2). The knockdown or overexpression efficiency was evaluated by qRT-PCR analysis.

### 2.7. Quantitative Real-Time Polymerase Chain Reaction (qRT-PCR)

The cellular total RNA was extracted with Trizol Reagent according to the manufacturer's instructions. Then, the total RNA was synthesized cDNA by using PrimeScript RT reagent Kit with gDNA Eraser (Takara, RR047A), and real-time PCR was conducted using one step SYBR® PrimeScript™ RT-PCR Kit II (Takara Bio Inc., Otsu, Japan). The gene expression levels were normalized to endogenous control *β*-Actin. All real-time PCR primer sequences were as follows: lnc-OAD (sense: AGGCTACCACAGCAGGCAAT, antisense: GAACGCACGGATGGAGGAT); Runx2 (sense: TGTTCTCTGATCGCCTCAGTG, antisense: CCTGGGATCTGTAATCTGACTCT); Osterix (sense: CCCCTTGTCGTCATGGTTACAG, antisense: AGAGAAAGCCTTTGCCCACCTA); *β*-actin (sense: GCCAACCGTGAAAAGATGAC, antisense: ACCAGAGGCATACAGGGACAG).

### 2.8. Western Blot Analysis

Western blot analysis was carried out as our previously described [19]. Briefly, cells were lysed with RIPA for 30 min at 4°C and centrifuged at 12,000 rpm for 15 min, and then, protein extractions were boiled for 5 min at 95°C in loading buffer. The samples were subjected to 10% SDS-PAGE and transferred to PVDF membranes, followed by incubating primary antibodies overnight at 4°C. Then, the blots were probed with the corresponding HRP-conjugated secondary antibodies for 1 hour at room temperature and visualized using electrochemiluminescence (ECL; Millipore, Darmstadt, Germany). The band signal intensities were quantified by ImageJ software (https://imagej.nih.gov/ij/).

### 2.9. Statistical Analysis

All data were expressed as mean ± SEM for each group with three biological replicates. Differences between groups were analyzed using Student's *t*-test or one-way analysis of variance followed by Dunnett's comparison test (for equal variance) or Dunnett's T3 comparison test (for unequal variance), and *P* < 0.05 was considered statistically significant.

## 3. Results

### 3.1. Lnc-OAD Was High Expression in MC3T3-E1 Preosteoblast Cells

**Figure 1:**
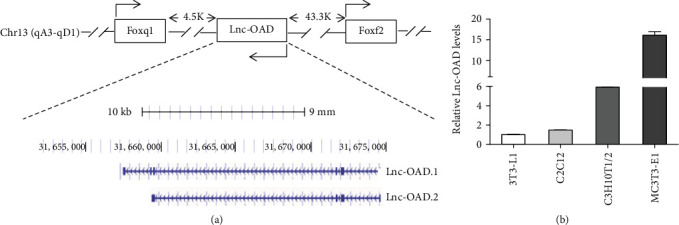
The chromosome location of lnc-OAD gene and its expression in different cell lines. (a) Schematic representation of the chromosomal location of lnc-OAD gene locus in the UCSC Genome Browser. Arrowheads indicate the orientation of transcription. (b) The expression levels of lnc-OAD in mouse different cell lines were evaluated by qRT-PCR.

To better understand the function of lnc-OAD, we carried out genome location analysis and detected its expression in mouse cell lines from different tissue sources. In the UCSC Genome Browser on NCBI37/mm9 Assembly, lnc-OAD, which transcribed from 1700018A04Rik gene, was mainly labeled as ENSMUST00000150418/NR_029439.1 or ENSMUST00000126138, and we named them as lnc-OAD.1 and lnc-OAD.2, respectively. Lnc-OAD was flanked by the Forkhead box Q1 (FOXQ1) and Forkhead box F2 (FOXF2) loci (Figure 1(a)). qRT-PCR results showed that lnc-OAD was highly expressed in MC3T3-E1 preosteoblast cells, but relatively low in 3T3-L1 preadipocyte cells and C2C12 myoblast cells (Figure 1(b)). We speculated that lnc-OAD might play an important role in osteoblast differentiation.

### 3.2. Lnc-OAD Expression Was Increased during BMP-2-Induced Osteoblast Differentiation

**Figure 2:**
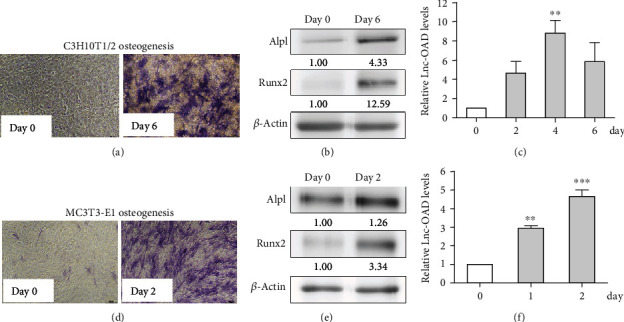
The dynamic expression of lnc-OAD during BMP-2-induced osteoblast differentiation of C3H10T1/2 and MC3T3-E1 cells. C3H10T1/2 cells (a–c) and MC3T3-E1 cells (d–f) were cultured with 200 or 100 ng/mL BMP-2 for the indicated days, respectively, alkaline phosphatase (ALP) staining was performed and representative images were shown ((a) C3H10T1/2; (d) MC3T3-E1), the osteogenic markers Runx2 and ALPL were detected by Western blot ((b) C3H10T1/2; (e) MC3T3T-E1), and the expression levels of lnc-OAD were detected by qRT-PCR at given time points during differentiation ((c) C3H10T1/2; (f) MC3T3-E1). The data are represented as means ± SEM (*n* = 3). ^∗∗^*P* < 0.01, ^∗∗∗^*P* < 0.001 vs. day 0 control.

We previously performed lncRNA microarray analysis and identified a series of differentially expressed lncRNAs during BMP-2-induced osteoblast differentiation in mouse C3H10T1/2 mesenchymal stem cells. Among them, lnc-OAD was one of the upregulated expression lncRNAs. To determine the role of lnc-OAD in BMP-2-induced osteoblast differentiation, we detected its expression level using quantitative RT-PCR. As shown in Figures 2(a) and 2(d), treatment with 200 ng/mL BMP-2 in C3H10T1/2 cells or 100 ng/mL BMP-2 in MC3T3-E1 cells obviously induced ALP staining in 6 or 2 days, respectively. Then, the expression of osteoblast differentiation markers ALP and RUNX2 were analyzed by Western blot. As shown in Figures 2(b) and 2(e), osteogenic markers ALP and RUNX2 were significantly increased, indicating successful induction of osteoblast differentiation by BMP-2. Furthermore, qRT-PCR validated the remarkable increases of lnc-OAD in both C3H10T1/2 and MC3T3-E1 cells following BMP-2 treatment (Figures 2(c) and 2(f)), suggesting that lnc-OAD might be essential for osteoblast differentiation.

### 3.3. Lnc-OAD Knockdown Significantly Suppressed BMP-2-Induced Early Osteoblast Differentiation

**Figure 3:**
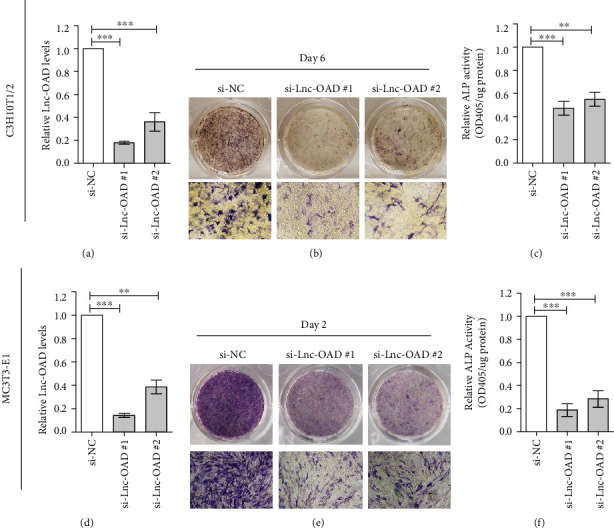
Lnc-OAD knockdown suppressed early osteoblast differentiation. (a, d) The cells were transfected with si-NC or si-Lnc-OAD, and the lnc-OAD expression levels were assayed by real-time PCR ((a) C3H10T1/2; (d) MC3T3-E1). After transfected with indicated siRNA for 24 h, C3H10T1/2 or MC3T3-E1 cells were cultured in BMP-2 differentiation medium for indicated times and then the ALP staining assays ((b) C3H10T1/2; (e) MC3T3-E1) and quantified ALP activities ((c) C3H10T1/2; (f) MC3T3-E1) were performed. Data were presented as mean ± SEM, *n* = 3, ^∗^*P* < 0.05, ^∗∗^*P* < 0.01, ^∗∗∗^*P* < 0.001 compared with NC.

To clarify the potential biological role of lnc-OAD in osteoblast differentiation, we knocked down lnc-OAD using two different sets of siRNAs targeting lnc-OAD (si#1 and si#2). As shown in Figures 3(a) and 3(d), treatment with two siRNA sequences resulted in marked decreases of lnc-OAD mRNA on day 2 posttransfection in C3H10T1/2 and MC3T3-E1 cells. Then, the cells transfected with siRNAs were induced osteoblast differentiation using BMP-2. According to the results of ALP staining (Figures 3(b) and 3(e)), the early osteoblast differentiation was inhibited significantly after transfection with siRNAs. As expected, osteoblast ALP activity was also significantly decreased (Figures 3(c) and 3(f)).

### 3.4. Overexpressing Lnc-OAD Increased BMP-2-Induced Early Osteoblast Differentiation

**Figure 4:**
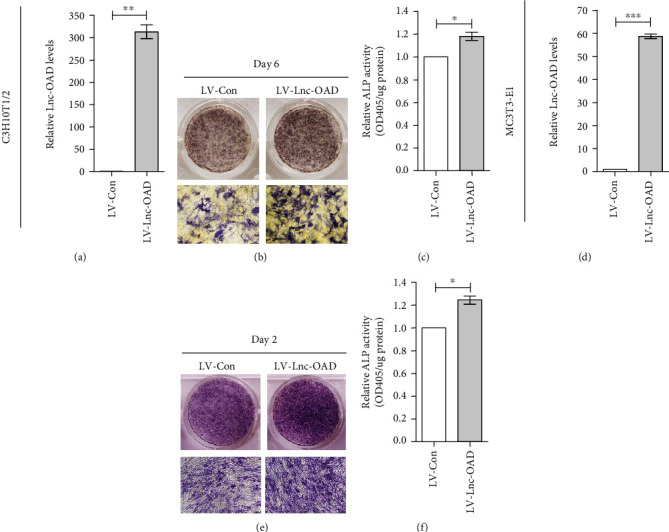
Lnc-OAD overexpression increased early osteoblast differentiation. (a, d) C3H10T1/2 and MC3T3-E1 cells were infected with LV-Con or LV-Lnc-OAD lentivirus vectors and selected using puromycin, and the overexpression efficiency was evaluated by qRT-PCR analysis. (b–f) Stable overexpression Lnc-OAD of C3H10T1/2 or MC3T3-E1 cells were cultured in BMP-2 differentiation medium for indicated times, and then, the ALP staining assays ((b) C3H10T1/2; (e) MC3T3-E1) and quantified ALP activities ((c) C3H10T1/2; (f) MC3T3-E1) were performed. Data were presented as mean ± SEM, *n* = 3, ^∗^*P* < 0.05, ^∗∗^*P* < 0.01, ^∗∗∗^*P* < 0.001 compared with LV-Con.

To further determine the effect of lnc-OAD in osteoblast differentiation, we generated stably overexpressed lnc-OAD C3H10T1/2 and MC3T3-E1 cell lines using lentiviral vectors. qRT-PCR confirmed dramatically elevated expression of lnc-OAD mRNA in C3H10T1/2 (Figure 4(a)) and MC3T3-E1 cells (Figure 4(d)). As expected, overexpression of lnc-OAD increased BMP-2-induced ALP staining and ALP activity in both C3H10T1/2 (Figures 4(b) and 4(c)) and MC3T3-E1 cells (Figures 4(e) and 4(f)). Taken together, these results suggest that lnc-OAD has the effects on stimulating BMP-2-induced osteoblast differentiation.

### 3.5. Lnc-OAD Was Required for BMP-2-Induced Late Osteoblast Differentiation and Matrix Mineralization of MC3T3-E1 Cells

**Figure 5:**
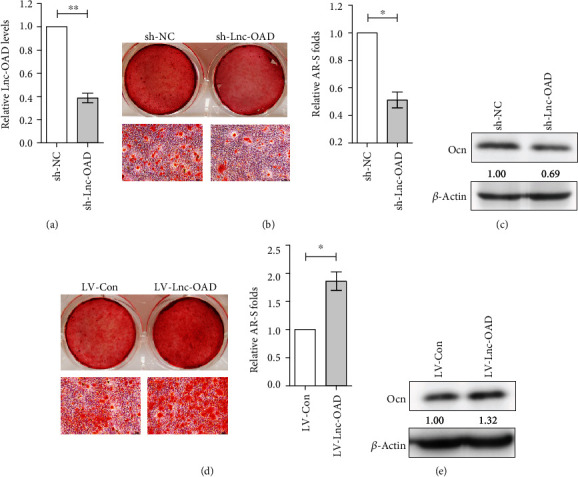
Lnc-OAD was required for BMP-2-induced late osteoblast differentiation and matrix mineralization of MC3T3-E1 cells. (a) MC3T3-E1 was infected with shRNA lentivirus vectors and selected using puromycin, and the knockdown efficiency was evaluated by qRT-PCR analysis. sh-NC and sh-Lnc-OAD MC3T3-E1 cells (b, c), LV-Con and LV-Lnc-OAD MC3T3-E1 cells (d, e) were cultured in 100 ng/mL BMP-2 differentiation medium containing AA and *β*-GP for 10 days, then the cells were stained with Alizarin red S solution (AR-S) and the AR-S content was measured (b, d), and the protein level of OCN was determined by western blot analysis (c, e). Data were presented as mean ± SEM, *n* = 3, ^∗^*P* < 0.05, ^∗∗^*P* < 0.01 compared with sh-NC or LV-Con.

Next, we examined whether lnc-OAD was required for late osteoblast differentiation. According to the Alizarin Red S staining, the lnc-OAD gene stably knockdown cells showed a drastic decrease in mineralized nodule formation (Figures 5(a) and 5(b)), while there was an increased mineralization nodule formation in MC3T3-E1 cells of stably overexpressed lnc-OAD (Figure 5(d)). Similarly, we also observed a downregulated OCN protein expression in lnc-OAD gene stably knockdown MC3T3-E1 cells (Figure 5(c)), whereas upregulated OCN protein expression in lnc-OAD gene stably overexpressed MC3T3-E1 cells (Figure 5(e)), which further suggesting that lnc-OAD is required for late osteoblast differentiation.

### 3.6. Lnc-OAD Regulated BMP-2 Induced the Expression of Osteogenic Transcription Factor Osterix

**Figure 6:**
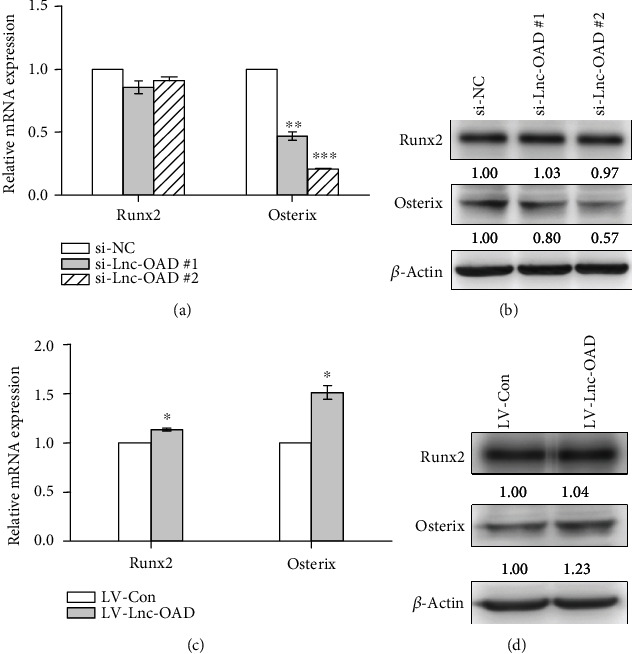
Lnc-OAD affected the Osterix expression level. After knockdown or overexpression of lnc-OAD, C3H10T1/2 cells were cultured in 200 ng/mL BMP-2 differentiation medium for 2 days, and then, the expression levels of osteoblast differentiation related transcription factors were analyzed by real-time PCR (a, c) or by Western blotting (b, d). Data were presented as mean ± SEM, *n* = 3, ^∗^*P* < 0.05, ^∗∗^*P* < 0.01, ^∗∗∗^*P* < 0.001, compared with si-NC or LV-Con.

Osteoblast differentiation was coregulated by a series of important transcription factors such as Runt-related transcription factor 2 (Runx2) and zinc finger transcription factor Osterix. To further investigate the mechanism by which lnc-OAD might regulate osteoblast differentiation, we detected the expression of above important transcription factors in osteogenic differentiation. As shown in Figure 6(a), the change of Osterix mRNA was most obvious after knockdown lnc-OAD. Next, we performed Western blot analysis to examine whether the protein level of Osterix was modulated by lnc-OAD. Consistent with the above mRNA results, knockdown lnc-OAD significantly decreased BMP-2-induced Osterix protein expression (Figure 6(b)). Whereas Osterix mRNA and Osterix protein expression were increased after overexpressed lnc-OAD (Figures 6(c) and 6(d)). However, no obvious changes of Runx2 protein expression were detected in knockdown or overexpressed lnc-OAD cells. Given that previous study has been proved that Osterix played a pivotal role in osteoblast differentiation [20], our data suggested that lnc-OAD might function as a regulator of BMP-2-induced osteoblast differentiation through fine-tuning the expression of Osterix.

### 3.7. Lnc-OAD Regulated AKT Signaling in BMP-2-Induced Osteoblast Differentiation

**Figure 7:**
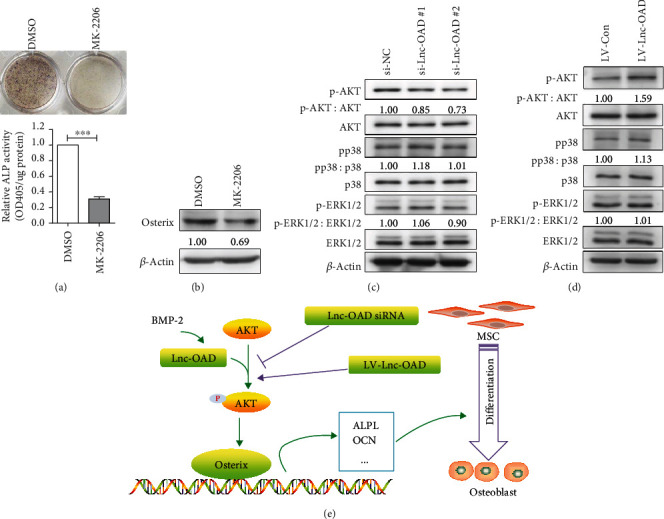
lnc-OAD affected AKT-Osterix signaling axis in BMP-2-induced osteoblast differentiation. (a, b) The C3H10T1/2 cells were cultured in 200 ng/mL BMP-2 differentiation medium in combination with DMSO (control) or MK-2206 (10 *μ*M); ALP staining and activity were performed on day 4 (a), and the protein expression level of Osterix was analysed on day 2 (b). (c) The C3H10T1/2 cells were transfected with indicated siRNA for 24 h and were subsequently treated with 200 ng/mL BMP-2 for 30 min, then the cell lysates were subjected to western blot analysis with indicated antibodies. (d) The LV-Con and LV-Lnc-OAD cells were treated with 200 ng/mL BMP-2 for 30 min, then the cell lysates were subjected to Western blot analysis with indicated antibodies. (e) Proposed mechanism for lnc-OAD in regulating BMP-2-induced osteoblast differentiation. Data were presented as mean ± SEM, *n* = 3, ^∗∗∗^*P* < 0.001, compared with DMSO control.

Given that previous studies have demonstrated that AKT signaling pathway was required for BMP-2-induced osteoblast differentiation and AKT pathway could regulate the expression of Osterix [21, 22], in addition, the drugs that inhibited AKT phosphorylation could also inhibit BMP-2-induced osteoblast differentiation [23]. We asked whether lnc-OAD regulates the AKT pathway. To investigate the potential role of endogenous AKT in BMP-2-induced osteoblast differentiation, we treated the C3H10T1/2 mesenchymal stem cells with AKT inhibitor and BMP-2. As shown in Figures 7(a) and 7(b), treatment with 10 *μ*M AKT inhibitor MK-2206 clearly decreased the ALP staining and the protein expression of Osterix induced by BMP-2, which further confirmed that AKT signaling pathway activation was necessary for BMP-2-induced osteoblast differentiation. Next, we examined whether the AKT phosphorylation induced by BMP-2 was modulated by lnc-OAD, as shown in Figures 7(c) and 7(d), knockdown lnc-OAD decreased, while overexpressed lnc-OAD increased, BMP-2-induced AKT phosphorylation. However, there were no obvious effects on other osteogenic signaling pathways such as pp38 and p-ERK1/2.

Thus, based on all current data, we proposed a model to explain the possible mechanism of lnc-OAD regulating BMP-2-induced osteoblast differentiation as follows (Figure 7(e)): BMP-2 was able to upregulate the expression of long noncoding RNA lnc-OAD, which led to the increase of AKT phosphorylation level and then activated the AKT pathway to upregulate the transcription factor Osterix. Finally, Osterix, possibly in combination with other factors, initiated the expression of downstream osteogenic differentiation specific genes such as ALP and OCN. In contrast, during this process, lnc-OAD siRNA prevented AKT phosphorylation whereas LV-lnc-OAD promoted AKT phosphorylation, which would eventually affected osteogenic differentiation.

## 4. Discussion

BMP-2, a member of the BMPs subfamily and secreted by osteoblasts, has a promoting effect on osteoblast differentiation [24]. In the present study, we found for the first time that lnc-OAD function was related to BMP-2-induced osteoblast differentiation. We showed that lnc-OAD expression was apparently increased during BMP-2-induced osteoblast differentiation in C3H10T1/2 and MC3T3-E1 cells, and knockdown of lnc-OAD markedly blocked, whereas overexpression of lnc-OAD promoted, BMP-2-induced early and late osteoblast differentiation. To further clarify the mechanisms though which lnc-OAD regulated osteoblast differentiation, we also revealed that lnc-OAD significantly affected the activity of the AKT-Osterix signaling axis. Thus, our data demonstrated for the first time that lnc-OAD was an important regulator in vitro via regulating AKT-Osterix signaling axis.

Many lines of evidence suggested that osteoblast differentiation was precisely controlled by a series of transcription factors such as Runx2, Osterix, Dlx2, Dlx5, and Atf4 [6, 25]. Among them, Runx2 and Osterix were considered as master regulators of the osteoblast differentiation, and both of them were regulated by BMP-2 [26, 27]. Runx2 regulated mesenchymal stem cells into an osteoblastic lineage, whereas inhibited differentiation into the lipogenic lineages [28]. Osterix acted downstream of Runx2 and was sufficient to activate some osteoblast differentiation genes [20]. In Osterix null mice, there was no bone formation [20]. Our study has shown that lnc-OAD significantly affected the osteoblast differentiation of C3H10T1/2 and MC3T3-E1 cells. Was this effect related to the above important regulatory factors? To address this issue, we analyzed the expression levels of the above transcription factors using real-time PCR and found BMP-2-induced Osterix expression was significantly regulated by lnc-OAD, which was consistent with the result of Osterix protein expression. Unexpectedly, the protein expression level of Runx2 did not show significant differences. Previous studies have demonstrated that Runx2 activation was controlled by many posttranscriptional regulations, such as acetylation and phosphorylation [29–31]. Further studies were needed to address whether lnc-OAD affected Runx2 posttranscriptional activation. Taken together, our study suggested that lnc-OAD affected BMP-2-induced osteoblast differentiation, at least in part, via regulating Osterix expression.

Previous studies have demonstrated that BMP-2 could regulate multiple signaling pathways inducing osteoblast differentiation such as AKT, MAPK, and Smad [1, 9]. PI3K/AKT pathway, an intracellular important signaling pathway, was required in all phases of osteoblast differentiation and maturation and inhibition of AKT activity impaired osteoblast development and function [9]. It was proved that AKT phosphorylates was required for BMP-2-induced osteoblast differentiation [21]. Indeed, we confirmed that BMP-2-induced ALP activity could be markedly inhibited after treatment with AKT inhibitor MK-2206. The present study further demonstrated that BMP-2-induced AKT phosphorylates were significantly suppressed by knockdown of lnc-OAD, whereas increased by overexpression of lnc-OAD. Considering that BMP-2 increased the protein level of transcription factor Osterix in an AKT activity-dependent manner [22] and that knockdown of Lnc-OAD not only inhibited AKT activity but also reduced Osterix expression level, it was therefore highly possible that lnc-OAD modulated BMP-2-induced osteoblast differentiation via regulating AKT-Osterix signal axis.

## 5. Conclusions

In summary, the present study showed that the newly identified long noncoding RNA lnc-OAD was required for BMP-2-induced osteoblast differentiation. Furthermore, the AKT-Osterix signal axis was identified as an important mechanism of lnc-OAD regulating BMP-2-induced osteoblast differentiation. These results might be helpful to further understand the regulation of osteogenic differentiation network by long noncoding RNA. However, further in vivo study was still required to fully elucidate the role of lnc-OAD in osteoblast differentiation and bone development.

## Data Availability

All data included in this study are available upon request through contacting the corresponding author.
